# HCN4 pacemaker channels attenuate the parasympathetic response and stabilize the spontaneous firing of the sinoatrial node

**DOI:** 10.1113/JP275303

**Published:** 2018-02-06

**Authors:** Yuko Kozasa, Noriyuki Nakashima, Masayuki Ito, Taisuke Ishikawa, Hiroki Kimoto, Kazuo Ushijima, Naomasa Makita, Makoto Takano

**Affiliations:** ^1^ Department of Physiology Kurume University School of Medicine 67 Asahi‐Machi Kurume 830‐0011 Japan; ^2^ Department of Anesthesiology Kurume University School of Medicine 67 Asahi‐Machi Kurume 830‐0011 Japan; ^3^ Department of Biochemistry and Cellular Biology, National Institute of Neuroscience NCNP Tokyo 187‐8502 Japan; ^4^ Department of Molecular Physiology, Nagasaki University Graduate School of Biomedical Sciences 1‐12‐4, Sakamoto Nagasaki 852‐8523 Japan

**Keywords:** HCN4 channels, parasympathetic nerve, bradycardia, sinoatrial node

## Abstract

**Key points:**

- The contribution of HCN4 pacemaker channels in the autonomic regulation of the sino‐atrial node (SAN) has been a matter of debate.
- The transgenic overexpression of HCN4 did not induce tachycardia, but reduced heart rate variability, while the conditional knockdown of HCN4 gave rise to sinus arrhythmia.
- The response of the SAN to β‐adrenergic stimulation was not affected by overexpression or knockdown of HCN4 channels.
- When HCN4 channels were knocked down, the parasympathetic response examined by cervical vagus nerve stimulation (CVNS) was enhanced; the CVNS induced complete sinus pause. The overexpression of HCN4 attenuated bradycardia induced by CVNS only during β‐adrenergic stimulation.
- We concluded that HCN4 pacemaker channels stabilize the spontaneous firing by attenuating the parasympathetic response of the SAN.

**Abstract:**

The heart rate is dynamically controlled by the sympathetic and parasympathetic nervous systems that regulate the sinoatrial node (SAN). HCN4 pacemaker channels are the well‐known causative molecule of congenital sick sinus syndrome. Although HCN4 channels are activated by cAMP, the sympathetic response of the SAN was preserved in patients carrying loss‐of‐function mutations of the HCN4 gene. In order to clarify the contribution of HCN4 channels in the autonomic regulation of the SAN, we developed novel gain‐of‐function mutant mice in which the expression level of HCN4 channels could be reversibly changed from zero to ∼3 times that in wild‐type mice, using tetracycline transactivator and the tetracycline responsive element. We recorded telemetric ECGs in freely moving conscious mice and analysed the heart rate variability. We also evaluated the response of the SAN to cervical vagus nerve stimulation (CVNS). The conditional overexpression of HCN4 did not induce tachycardia, but reduced heart rate variability. The HCN4 overexpression also attenuated bradycardia induced by the CVNS only during the β‐adrenergic stimulation. In contrast, the knockdown of HCN4 gave rise to sinus arrhythmia, and enhanced the parasympathetic response; complete sinus pause was induced by the CVNS. *In vitro*, we compared the effects of acetylcholine on the spontaneous action potentials of single pacemaker cells, and found that similar phenotypic changes were induced by genetic manipulation of HCN4 expression both in the presence and absence of β‐adrenergic stimulation. Our study suggests that HCN4 channels attenuate the vagal response of the SAN, and thereby stabilize the spontaneous firing of the SAN.

## Key points

- The contribution of HCN4 pacemaker channels in the autonomic regulation of the sino‐atrial node (SAN) has been a matter of debate.
- The transgenic overexpression of HCN4 did not induce tachycardia, but reduced heart rate variability, while the conditional knockdown of HCN4 gave rise to sinus arrhythmia.
- The response of the SAN to β‐adrenergic stimulation was not affected by overexpression or knockdown of HCN4 channels.
- When HCN4 channels were knocked down, the parasympathetic response examined by cervical vagus nerve stimulation (CVNS) was enhanced; the CVNS induced complete sinus pause. The overexpression of HCN4 attenuated bradycardia induced by CVNS only during β‐adrenergic stimulation.
- We concluded that HCN4 pacemaker channels stabilize the spontaneous firing by attenuating the parasympathetic response of the SAN.

## Introduction

Heart rate (HR) regulation is essential for the homeostasis that ensures appropriate cardiac output under various physiological conditions. The HR is determined by spontaneous action potentials (SAPs) of cardiac pacemaker cells in the sinoatrial node (SAN). The automaticity of pacemaker cells originates from the slow diastolic depolarization phase. Sympathetic and parasympathetic nerve stimulations change the slope of the slow diastolic depolarization in opposite directions, thereby regulating the firing frequency of pacemaker cells (Mangoni & Nargeot, 2008).

The slow diastolic depolarization, also called the pacemaker potential, is generated by two synergic mechanisms: the calcium clock and the membrane clock. In the former mechanism, spontaneous Ca^2+^ release from the sarcoplasmic reticulum activates an inward Na^+^–Ca^2+^ exchanger current (Lakatta & DiFrancesco, 2009; Lakatta *et al*. 2010). The latter mechanism comprises the sequential activation of voltage‐gated inward channels, the hyperpolarization‐activated cyclic nucleotide gated channels (HCN1, HCN2 and HCN4), low‐threshold calcium channels (Cav3.2) and dihydropyridine‐sensitive calcium channels (Cav1.2 and Cav1.3), on the plasma membrane. The latter mechanism comprises the sequential activation of voltage‐gated inward channels on the plasma membrane; the hyperpolarization‐activated cyclic nucleotide gated channels (HCN1, HCN2 and HCN4), low‐threshold calciun channels (Cav3.2) and dihydropyridine‐sensitive calcium channels (Cav1.2 and Cav1.3) (Mangoni *et al*. 2003, 2006; Bucchi *et al*. 2012; Herrmann *et al*. 2012; Mesirca *et al*. 2015).

In the autonomic regulation of the pacemaker potential, the specific impacts of these molecules are still a matter of debate. Amongst them, the ‘funny’ current (*I*_f_) has been suggested to play a pivotal role because *I*_f_ is activated by cAMP (DiFrancesco, 2010). However, loss‐of‐function studies of HCN4, a major subtype responsible for *I*_f_ in the SAN, have demonstrated that HCN4 is not essential for tachycardia induced by β‐adrenergic stimulation: HCN4‐knockout mice developed variable degrees of dysfunction in pacemaker automaticity ranging from recurrent sinus pause to lethal bradycardia (Bucchi *et al*. 2012; Herrmann *et al*. 2012). Such pacemaker dysfunction was rescued by genetic ablation of G‐protein‐coupled inward rectifier K^+^ channels (GIRK4) that were activated by M2 muscarinic receptors in the SAN (Mesirca *et al*. 2015). Recent studies have demonstrated that changes in the expression levels of HCN channels in the SAN are correlated with basal HR; in pregnant mice, an increase in HCN2 expression in the SAN gave rise to tachycardia (El Khoury *et al*. 2013). Exercise training was shown to induce sinus bradycardia via down‐regulation of HCN4 expression in the SAN (D'Souza *et al*. 2014).

These findings suggest that changes in the expression level of HCN channels could modify autonomic response as well as basal HR, particularly parasympathetic response of the SAN in both positive and negative directions. To test this hypothesis, we employed both gain‐of‐function and loss‐of‐function approaches; using a TET‐off system, we generated novel transgenic mice in which the expression level of HCN4 could be changed from zero to ∼3 times that found in wild‐types (Nakashima *et al*. 2013). Using this system, we performed telemetric ECG recordings, heart rate variability (HRV) analysis, and cervical vagus nerve stimulation, as well as patch clamp experiments using single pacemaker cells. We demonstrate that HCN4 channels attenuate the parasympathetic response of the SAN, and stabilize the spontaneous firing of the SAN. Preliminary results have been communicated at the Physiological Society of Japan (Kozasa *et al*. 2016).

## Methods

### Ethical approval

All animal experiments were approved by the Animal Ethics Committee of Kurume University. Animal care and experiments conform to the guidelines of *The Journal of Physiology* (Grundy, 2015). We used 14‐ to 25‐week‐old mice of both sexes (body weight ∼20–25 g). After finishing *in vivo* experiments, the mice were deeply anaesthetized with 5.0% sevoflurane in a plastic chamber, and euthanized by quick decapitation.

### Generation of HCN4^+/tTA_TRE^ mouse

HCN4^+/tTA_TRE^ was generated as previously reported (Nakashima *et al*. 2013; Bond *et al*. 2000). HCN4^+/tTA_TRE^ mice were identified by genomic PCR using the forward primer (5′‐CGA ATA AGA AGG CTG GCT CTG CAC C‐3′) and the reverse primer (5′‐GAG CAG CCT ACA TTG TAT TGG CAT G‐3′). To inhibit HCN4 expression from the mutant allele, food pellets containing doxycycline (DOX; 6 mg g^−1^, Research Diet, NJ, USA) were provided *ad libitum*.

### Generation of HCN4^+/luc^ mouse

To create the 5′ and 3′ homologous arms of the targeting vector, C57BL/6J mouse genomic DNA containing the *Hcn4* gene was obtained from a BAC clone (clone ID: RP23‐324A14, Roswell Park Cancer Institute, Buffalo, NY, USA). The cDNA of a fusion protein of firefly luciferase (U_47298) and enhanced green fluorescent protein (EGFP; U_03453.1) followed by SV40 polyadenylation signal and a neomycin‐resistance gene cassette were inserted in‐frame into the start codon of the *Hcn4* gene. The targeting vector was linearized by a *Sal*I site and electrophoresed into C57BL/6J embryonic stem cells. The G418‐resistant colonies were picked up and screened for homologous recombination by PCR and Southern blot. Chimeras were generated by injecting the correctly targeted embryonic stem cells into blastocysts of Balb/c mice and were identified by coat colour. Chimeric male founder mice were mated to C57BL/6J female mice to generate F1 heterozygous mice for the knockin line. F1 heterozygous mice were crossed with CAG‐Cre mice to delete the neomycin cassette. The HCN4^+/luc^ mice were identified by PCR using the forward primer 5′‐CGA GCT GGA CGG CGA CGT AAA CGG C‐3′ and reverse primer 5′‐CAT CCT TAG GGA GAA TTT GTT GAC C‐3′.

### Quantitative PCR

Mice were deeply anaesthetized with 3.0% sevoflurane, and the whole hearts were quickly removed (*n* = 3 in each strain). Total RNA was obtained from the whole heart using Trizol reagent (Thermo Fisher Scientific, Waltham, MA, USA) following the manufacturer's instructions. Single strand cDNA was synthetized using Superscript III (Thermo Fisher Scientific). Quantitative real‐time reverse transcription polymerase chain reaction (qPCR) was performed using predesigned TaqMan Gene Expression assays probes (Thermo Fisher Scientific): mouse HCN1 (Mm00468832_m1), HCN2 (Mm00468538_m1), HCN3 (Mm01212852_m1) and HCN4 (Mm01176086_m1). Relative levels of mRNAs were normalized to the level of 18S rRNA.

### Microarray analysis

In each strain of mouse, the SAN tissue (1.5 × 0.5 mm) was collected from three hearts. Total RNA was isolated using an RNeasy Lipid Tissue Kit (Qiagen, Germantown, MD, USA). The RNA sample was processed for hybridization on a MouseGene 2.0ST Array (Affymetrix, Santa Clara, CA, USA) using a GeneChip WT Plus Reagent Kit (Thermo Fisher Scientific). Fold change > ±1.5 was regarded as a significant difference.

### Western blotting

Western blotting was performed in triplicate. Proteins were extracted from the whole heart and kidney of transgenic mice using T‐PER Tissue Protein Extraction Reagent (Thermo Fisher Scientific) following the manufacturer's instruction. Equal amounts of proteins were separated on 7% SDS‐polyacrylamide gels and transferred to nitrocellulose membranes (Thermo Fisher Scientific). The membranes were incubated with primary anti‐Hcn4 polyclonal antibody (1:200, Alomone Labs, Jerusalem, Israel), primary anti‐β‐actin polyclonal antibody (Ab) (1:1000, MBL, Nagoya, Japan), and goat‐anti rabbit IgG HRP‐Ab (1:1000, MBL), or anti‐α‐tubulin polyclonal Ab‐HRP‐DirecT (1:1000, MBL). Signals were visualized by Immobilon Western HRP Substrate (Millipore, Billerica, MA, USA) and FluorChem FC2 (R&D Systems, Minneapolis, MN, USA).

### Luminescence imaging

One hundred and fifty microlitres of XenoLight RediJect d‐luciferin (30 mg ml^−1^, ParkinElmer, Waltham, MA, USA) was injected into the peritoneal cavity of HCN4^+/luc^ mice. *In vivo* imaging was performed 15 min after the injection using IVIS‐100 (ParkinElmer) under general anaesthesia with 1.5% isoflurane. Thereafter, the hearts were removed under general anaesthesia with 3.0% isoflurane, and immersed in nominally Ca^2+^‐free Tyrode solution containing 30 μg ml^−1^ d‐luciferin. The right atrium and both ventricles were opened and fixed on silicone rubber. *In vitro* luminescence images were taken by an EM‐CCD camera (ImagEM2, Hamamatsu Photonics, Hamamatsu, Japan) on a macro zoom microscope (MV‐10; Olympus, Tokyo, Japan) or on a Slice scope (Scientifica, Uckfield, UK) with a water immersion objective lens (×40, NA = 0.8, Olympus).

### Isolation of single pacemaker cell and patch‐clamp recordings

After anaesthetizing with 3.0% sevoflurane, the SAN tissue was excised in pre‐warmed (36°C) normal Tyrode solution containing (in mm): NaCl 140, KCl 5.4, CaCl_2_ 1.8, MgCl_2_ 0.5, NaH_2_PO_4_ 0.33, HEPES 5 and glucose 5.5 (pH 7.4 with NaOH). The SAN tissue was enzymatically digested for 20 min at 37°C in nominally Ca^2+^ free Tyrode solution containing 344 U ml^−1^ liberase (Roche Molecular Systems, Pleasanton, CA, USA) and 1.9 U ml^−1^ elastase (Worthington Biochemical Corp., Lakewood, NJ, USA). Single pacemaker cells (PMCs) were mechanically dissociated from tissue strips in KB solution containing (in mm): glutamic acid 70, KCl 30, KOH 70, KH_2_PO_4_ 10, MgCl_2_ 1, taurine 20, EGTA 0.3, HEPES 10 and glucose 10 (pH 7.4 with KOH). Isolated PMCs were stored in KB solution at 4°C before experiments. The membrane current and the spontaneous action potentials (SAPs) were recorded using an Axopatch 200B amplifier (Molecular Device, Sunnyvale, CA, USA) at 33–35°C.

The *I*_f_ was recorded using the ruptured whole cell patch method in Tyrode solution containing 2 mmol l^−1^ BaCl_2_. The resistance of patch electrodes was 2–5 MΩ when filled with standard high K^+^ intracellular solution containing (in mm): aspartic acid 100, K_2_‐ATP 5, Na_2_‐creatine phosphate 5, EGTA 0.1, KCl 30, MgCl_2_ 1, CaCl_2_ 0.04 (pCa 7.0) and HEPES 5 (pH 7.2 with KOH). The voltage‐dependent activation of *I*_f_ was evaluated with the two‐step pulse protocol; the amplitudes of the time‐dependent *I*_f_ current activated by the test pulse (−150 mV) were normalized by their maximal value, and were plotted against the preceding conditioning pulses (−50 to −140 mV). We then fitted Boltzmann's equation: % activation = 1/(1 + exp((*V*_1/2_ − *V*_m_)/*s*)), where *V*_1/2_ is the voltage for the half‐maximal activation; *V*_m_, membrane potential; *s*, slope factor. The diastolic depolarization rate (DDR) of SAPs was calculated as a slope between two points: the point where d*V*/d*t* = 0, and the point at two‐thirds of the diastolic depolarization.

### Telemetric ECG recording and HR histogram

Under general anaesthesia with 1.5% isoflurane, a telemetric transmitter (TA11ETA‐F10, DSI, St. Paul, MN, USA) was implanted into the subcutaneous pocket with electrodes arranged above and below the heart. After at least 8 days of recovery from the surgical stress, the ECG was recorded in a conscious, free‐moving condition. Mice were kept in an air‐conditioned room at 25°C under a 12 h light–12 h dark cycle. Food and water were provided *ad libitum*.

The analogue telemetric ECG signals were digitized at 1 kHz and recorded using PONEMAH software (DSI, USA). R peaks of the ECG signal were detected, and the mean HR was calculated from the RR interval and averaged for 1 min. Values of the mean HR were binned using 70 equally distributed windows (bin width; 10 beats min^−1^) in a range from 100 to 800 beats min^−1^.

### Analysis of the HRV

The HRV was analysed at the most frequently observed HR determined by the HR histogram during the low activity period. We performed time domain analysis including RR‐plot, Poincaré plot and frequency domain analysis. RR‐plot: series of the RR intervals were plotted for 60 s. Poincaré Plot: RR intervals (*n*; abscissa) were plotted against the following RR intervals (*n* + 1; ordinate). The total number of points was 20,000. Standard deviation of all RR intervals (SDNN) and root mean square of the difference of successive RR intervals (RMSSD): 2 h ECG traces were selected, and artefact correction was performed by excluding the data points outside the mean ± 2 SD. Frequency domain analysis: the RR‐plot was interpolated by cubic spline interpolation at 50 ms intervals to create equidistant points suitable for fast Fourier transformation (FFT) using Kubios HRV standard software (Kubios, Norra Savolax, Finland). After artefact correction excluding the data points outside the mean ± 2 SD, FFT was performed using 2048 points and a half‐overlap within a Hanning window, and the power spectral density was calculated.

### Vagal nerve stimulation

Control ECG (lead II) was recorded using PowerLab 26T (AD Instruments, Colorado Springs, CO, USA) for 5 min under general anaesthesia with 3.0% sevoflurane. Thereafter, the right cervical vagus nerve (CVN) was gently detached and ligated with an 8‐0 nylon suture. After the HR returned to control levels, the right CVN was stimulated at the peripheral side of the ligation with bipolar electrodes (10 V at 20 Hz for 10 s). Body temperature was monitored and kept at 37°C using a heating pad during the experiment. After finishing the experiments, the mice were deeply anaesthetized with 5.0% sevoflurane in a plastic chamber, and euthanized by quick decapitation.

### Data analysis

In molecular biological experiments, *n* denotes the number of samples and equals the number of animals used. In *in vivo* experiment, *n* is the number of the observations within a single experiment and equals the number of animals used. In patch clamp experiments, the membrane current was recorded in 14 cells isolated from six wild‐type (WT) mice, 19 cells from six overexpression (OEx) mice, and 17 cells from five knockdown (KD) mice. SAPs were recorded in 11 cells isolated from four WT mice, 11 cells from three OEx mice, 11 cells from four KD mice in Fig. 8; and in seven cells isolated from three WT mice, eight cells from three OEx mice, eight cells from three KD mice in Fig. 9. The parameters of SAP were averaged from five successive SAPs in each experimental condition. All data are presented as the mean ± SEM. For multiple comparisons, two‐way ANOVA followed by the Holm–Sidak test was used in Figs 8 and 9. One‐way ANOVA followed by Dunnett's test was used in all other experiment. Differences were considered significant when *P *< 0.05. Statistical analysis was performed using SigmaPlot 12.5 (Systat Software, San Jose, CA, USA).

## Results

### Generation of HCN4‐overexpression and HCN4‐knockdown mice

**Figure 1:**
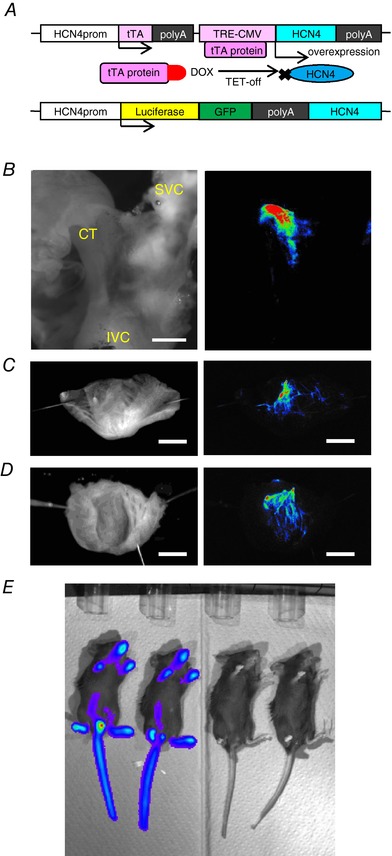
Generation of HCN4‐OEx, HCN4‐KD mice and luminescence imaging of HCN4^+/luc^ *A*, genetic structures of HCN4 knockin mice. Upper panel, schematic illustration of a genetic switch. tTA, tetracycline transactivator followed by termination signal SV‐40 polyA. TRE‐CMV, tetracycline‐responsive element fused with a cytomegalovirus minimal promoter. Lower panel, firefly luciferase cDNA fused with GFP cDNA followed by SV‐40 polyA. *B–D*, *ex vivo* imaging of cardiac conduction system. Left panels, bright field. Right panels, luminescence imaging of the same preparations as left panels. The intensity of luminescence signal is shown by a pseudocolour spectrum. *B*, the right atrium preparation. CT, crista terminalis; IVC, inferior vena cava; SVC, superior vena cava. Scale bar, 0.5 mm. *C*, the intraluminal surface of the right ventricle. Scale bar, 2 mm. *D*, the intraluminal surface of the left ventricle. Scale bar, 2 mm. *E*, *in vivo* luminescence imaging; left, 2 male mice, HCN4^+/luc^; right, 2 male mice, WT littermates. Exposure time of luminescence imaging in *B–E* was 3 min.

To overexpress HCN4 channels at their physiological expression locus, we inserted a genetic switch using a tetracycline transactivator (tTA)/operator system upstream of the translation initiation site of the HCN4 gene as depicted in Fig. 1 *A* (Bond *et al*. 2000; Nakashima *et al*. 2013). In this system, the HCN4‐promoter drives the expression of tTA protein, which activates the transcription of HCN4 from the tetracycline responsive element (TRE)–CMV promoter with high efficiency. Furthermore, the transcription of HCN4 could be completely suppressed by doxycycline (DOX), which interferes with the binding of tTA to TRE. Therefore, in homozygous HCN4^tTA_TRE/tTA_TRE^ mice, it should theoretically be possible to regulate the expression level of HCN4 from zero to several times that of the WTs. However, we could not obtain homozygous HCN4^tTA_TRE/tTA_TRE^ mice, suggesting that excessive overexpression of HCN4 may be embryonically lethal for unknown reasons. We also applied DOX to HCN4^+/tTA_TRE^ mothers during pregnancy. However, we could not obtain HCN4^tTA_TRE/tTA_TRE^, probably because this mutant administrated DOX shows similar embryonic lethality to that reported in HCN4^−/−^ mice (Herrmann *et al*. 2007). Consequently, we used heterozygous HCN4^+/tTA_TRE^ mice as a model of HCN4‐overexpression in this study and designated them as overexpression (OEx) mice.

We also knocked‐in cDNA of a fusion protein of firefly luciferase and GFP into the translation initiation site of HCN4 (HCN4^+/luc^; Fig. 1 *A*). In this mouse, HCN4 expression from the mutant allele was completely inhibited, but the anatomical locus of HCN4 expression could be visualized with luminescence derived from the luciferin–luciferase reaction. As shown in Fig. 1 *B*, luminescence could be observed in the SAN area of right atrial preparations, which was similar to the findings in a previous study (Hoesl *et al*. 2008). As shown in Fig. 1 *C* and *D*, the luminescence signal was also detected in the Purkinje fibres. *In vivo*, intense luminescence was also observed, most likely derived from olfactory neurons, taste buds and osteoclasts in Fig. 1 *E* (Stevens *et al*. 2001; Nakashima *et al*. 2013; Notomi *et al*. 2015). However, GFP fluorescence was too weak and indistinguishable from the autofluorescence signal.

**Figure 2:**
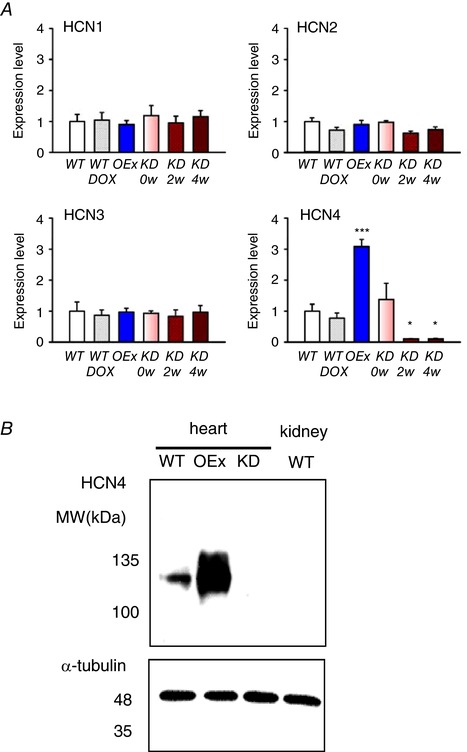
Expression levels of HCN4 were altered in OEx and KD mice *A*, qPCR for mRNAs of HCN1, 2, 3 and 4 were obtained from the whole hearts of WT (*n* = 4), OEx (*n* = 4) and KD (*n* = 4) mice. DOX was applied for 2 and 4 weeks, respectively. ^*^ *P *< 0.05, ^***^ *P *< 0.001. *B*, representative western blots for HCN4 and tubulin protein in the heart.

We next generated a double knockin mouse (HCN4^Luc/tTA_TRE^) by crossbreeding HCN4^+/tTA_TRE^ and HCN4^+/luc^ mice. The likelihoods of all possible genotypes were at normal Mendelian rates. All strains of HCN4^+/luc^, HCN4^+/tTA_TRE^ and HCN4^Luc/tTA_TRE^ mice grew up normally without any apparent behavioural disorders. In order to knockdown the expression of the HCN4 protein, we administrated DOX to HCN4^Luc/tTA_TRE^ mice. To compare expression levels of HCN4 mRNA, we performed qPCR experiments. As shown in Fig. 2 *A*, the expression level of HCN4 was about three times higher in OEx mouse hearts than in the WTs and was almost zero in knockdown (KD) mice after administration of DOX for 2 or 4 weeks. A lack of the HCN4 protein in KD mice was also confirmed by western blots (Fig. 2 *B*). We therefore designated HCN4^Luc/tTA_TRE^ mice administered DOX as HCN4‐KD mice. On the other hand, expression levels of other members of the HCN family (HCN1, 2 and 3) were not altered in the hearts of OEx or KD mice (Fig. 2 *A*). We also carried out microarray analysis using mRNA isolated from the SAN of WT, OEx and KD mice; we confirmed no significant changes in the expression levels of such ion channels, transporters, receptors and related molecules that reportedly participate in the regulation of SAN function as summarized in Table 1 (Posokhova *et al*. 2013; Sah *et al*. 2013).

**Table 1:** Microarray analysis of transcripts potentially engaged in the regulation of pacemaker potential in SAN tissue

Public gene ID	Gene symbol	Fold‐change WT *vs*. OEx	Fold‐change WT *vs*. KD
NM_001164325	*Trpm7*	−1	1.11
NM_001253860	*Scn5a*	−1.4	−1.17
NM_001159533	*Cacna1c*	−1.24	−1.32
NM_001083616	*Cacna1d*	−1.18	−1.29
NM_001112813	*Cacna1g*	−1.06	−1.42
NM_001163691	*Cacna1h*	−1.29	−1.31
NM_001304810	*Kcnj3*	−1.25	−1.25
NM_001025584	*Kcnj6*	−1.16	−1.13
NM_008429	*Kcnj9*	−1.05	−1
NM_010605	*Kcnj5*	−1.06	−1.15
NM_008434	*Kcnq1*	−1.17	−1.1
NM_001294162	*Kcnh2*	−1.24	−1.12
NM_001112798	*Slc8a1*	−1.11	−1.18
NM_007504	*Atp2a1*	−1.31	−1.16
NM_001110140	*Atp2a2*	−1.05	−1.05
NM_009109	*Ryr1*	−1.11	−1.09
NM_023868	*Ryr2*	−1.28	−1.26
NM_009813	*Casq1*	1.15	−1.02
NM_009814	*Casq2*	−1.23	−1.08
NM_001174053	*Camk2b*	−1.05	−1.2
NM_001112697	*Chrm1*	−1.09	−1.12
NM_203491	*Chrm2*	−1.26	−1.27
NM_001282061	*Rgs6*	−1.09	−1.17
NM_001284380	*Adra1b*	−1.35	−1.43
NM_001271759	*Adra1a*	1.05	1.15
NM_025331	*Gng11*	1.01	1.1

Fold changes of the WT *vs*. OEx and WT *vs*. KD mice are shown. The negative values indicate that the amounts of transcripts were less than those of WTs. None of the transcripts were significantly down‐regulated or up‐regulated.

### ‘Funny’ current (*I*_f_) in pacemaker cells of transgenic mice

**Figure 3:**
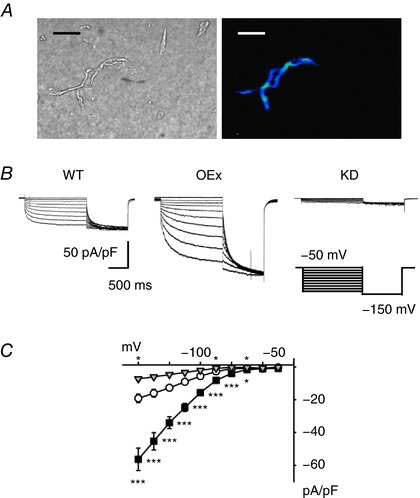
*I*_f_ currents in WT, OEx and KD pacemaker myocytes *A*, representative PMCs isolated from HCN4^+/luc^ mice. Right panel, bright field. Left panel, luminescence image taken by 5 min exposure. Note that luminescence signals were detectable only in ‘spindle shaped’ myocytes. Scale bar, 20 μm. *B*, representative *I*_f_ traces activated by voltage steps from −60 to −140 mV (10 mV increment). The holding potential was −50 mV. The scale bar indicates current density (pA/pF). *C*, *I–V* curves of the *I*_f_ in WT (open circles; *n* = 14), OEx (filled squares; *n* = 19), and KD (grey triangles; *n* = 17) mice. ^*^ *P *< 0.05, ^***^ *P *< 0.001.

We next compared the densities of ‘funny’ currents (*I*_f_) in pacemaker cells (PMCs) isolated from the SAN of WT, OEx and KD mice. We selected PMCs based on their morphology and motility (spindle cells and elongated cells with spontaneous beating). As shown in Fig. 3 *A*, we confirmed luminescence signals derived from these types of myocytes isolated from HCN4^+/luc^ mice. The membrane capacitance of the PMCs was not significantly different in WT (46.3 ± 6.9 pF, *n* = 14), OEx (42.3 ± 4.3 pF, *n* = 19) and KD (48.7 ± 6.4 pF, *n* = 17) mice. The amplitudes of the *I*_f_, defined as the time‐dependent current activated by the hyperpolarization, were normalized by the cellular capacitance and plotted in Fig. 3 *C*. In the PMCs of OEx mice, the densities of the *I*_f_ were significantly larger than those of the WTs between −130 and −70 mV (Fig. 3 *C*, filled squares; −130 to −80 mV: *P *< 0.001; −70 mV: *P = *0.028). The activation time course was also slower, indicating that in the PMCs of OEx mice, HCN4, i.e. the most slowly activating subtype, was robustly overexpressed. In KD mice, the density of the residual *I*_f_ was significantly smaller than that of the WTs at −130, −90 and −70 mV (Fig. 3 *C*, inverted triangles; *P = *0.013∼0.022). In this experiment, the amplitude of *I*_f_ may have been overestimated because we excluded quiescent PMCs that completely lacked an *I*_f_, and focused on irregularly beating PMCs, which expressed residual HCN1 and HCN2 at relatively higher densities. In line with this this notion, the residual *I*_f_ in KD mice showed a faster activation time course. These findings well agreed with previous reports demonstrating that the residual *I*_f_ of conditional HCN4 knockout mice was mainly due to HCN1 and HCN2, whose activation time kinetics are faster than that of HCN4 (Herrmann *et al*. 2007; Hoesl *et al*. 2008; Baruscotti *et al*. 2011).

Taken together, the changes in the density of the *I*_f_ in OEx and KD mice were consistent with the molecular biological results shown in Fig. 1.

### HR histogram was altered in telemetric ECG recordings of mutant mice

**Figure 4:**
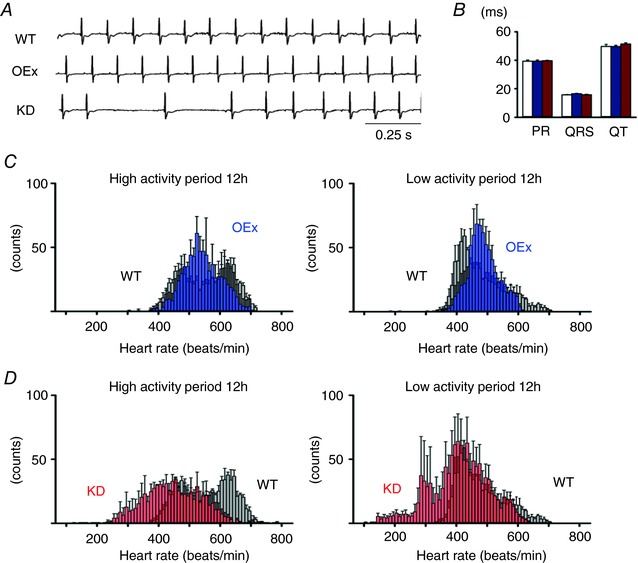
Telemetric ECG recordings and HR histograms of free‐moving WT, OEx and KD mice *A*, representative ECG traces of WT, OEx and KD mice. *B*, summary of ECG parameters for WT (white, *n* = 3), OEx (blue, *n* = 3) and KD (red, *n* = 3) mice. *C*, HR histograms of WT (grey, *n* = 3) and OEx (blue, *n* = 3) mice. A histogram was constructed for the 12 h low activity period (right panel) and the 12 h high activity period (left panel). Bin width is 10 beats min^−1^. *D*, HR histograms of WT (grey, *n* = 3) and KD (red, *n* = 3) mice, as in *C*.

Based on the above findings, we next investigated the phenotypic changes *in vivo* by recording telemetric ECG in freely moving, conscious WT, OEx and KD mice. Contrary to our expectation, no apparent tachycardia was observed in OEx mice. KD mice started to exhibit sinus arrhythmia 4–5 days after starting DOX administration, and reduction of the HR gradually proceeded. As demonstrated in Fig. 2 *A*, suppression of HCN4 expression reached a steady state 2 weeks after DOX administration. We therefore performed 24 h telemetric ECG recordings at this time point. Fig. 4 *A* shows typical ECG traces recorded during the low activity period; both WT and OEx mice showed normal sinus rhythms, whereas KD mice showed severe sinus arrhythmia. As summarized in the bar graphs (Fig. 4 *B*), PR, QRS and QT intervals were not significantly different, suggesting that electrical conduction in the atrioventricular node (AVN) and in Purkinje fibres was not changed in KD or OEx mice.

In contrast, SAN function represented by HR histograms was clearly different between WT (*n* = 3, grey bars), OEx (*n* = 3, blue) and KD (*n* = 3, red) mice, as shown in Fig. 4 *C* and *D*. In the WTs, an HR histogram during the high activity period showed two peaks: a slow peak at ∼480–500 beats min^−1^ and a fast peak at ∼620–640 beats min^−1^. During a low activity period, the fast peak became ambiguous, and only a single, broad peak at ∼440–460 beats min^−1^ became evident. The fluctuation range of the HR (difference between the minimum and maximum HR) was 330.0 ± 12.3 beats min^−1^ in the high activity period and 338.7 ± 16.5 beats min^−1^ in the low activity period. These patterns were similar to the results reported in a previous study (Alig *et al*. 2009; Fenske *et al*. 2013).

In OEx mice, the HR histogram in the high activity period showed a single, broad peak at ∼540–560 beats min^−1^, and the fluctuation range was 322.7 ± 32.2 beats min^−1^ (Fig. 4 *C*). In the low activity period, the HR histogram also showed a single peak at ∼460–480 beats min^−1^, and the fluctuation range was 265.7 ± 33.2 beats min^−1^, which was smaller than that of the WTs. As OEx mice showed no apparent behavioural alterations, these changes appeared due to SAN function, rather than physical activity.

In KD mice, the entire HR histograms shifted to lower values, and the fluctuation range of the HR was larger than that of the WTs, both in the high and low activity periods (Fig. 4 *D*). These findings were in agreement with previous reports on HCN1‐, HCN2‐ and HCN4‐knockout mice (Ludwig *et al*. 2003; Herrmann *et al*. 2007; Baruscotti *et al*. 2011; Fenske *et al*. 2013). It should be noted in particular that during the low activity period, KD mice showed severe bradycardia (∼100–200 beats min^−1^) due to a recurrent sinus pause, which was most probably due to the circadian change of autonomic nerve activity.

### Beat‐to‐beat HR variability was reduced by HCN4 overexpression and enhanced by HCN4 knockdown

The telemetric ECG recordings in the previous section suggested that overexpression of HCN4 as well as knockdown of HCN4 could affect autonomic regulation of SAN function. Therefore, we next analysed the beat‐to‐beat HR variability (HRV) in WT, OEx and KD mice, focusing on the low activity period during which parasympathetic nerve activity was predominant.

**Figure 5:**
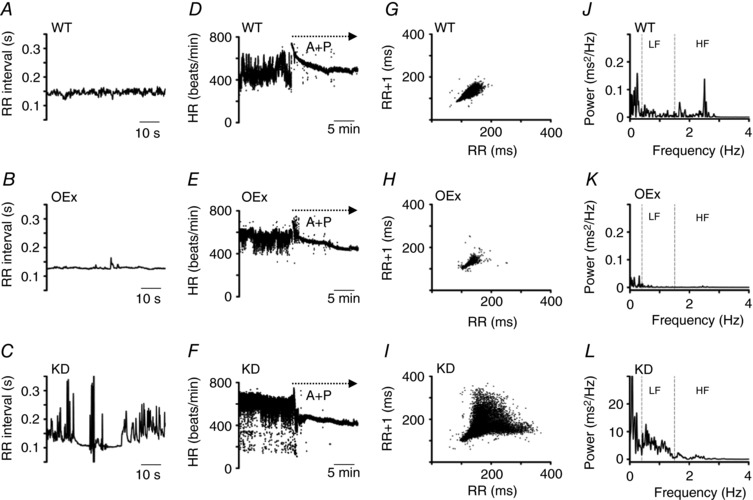
Analysis of HRV in WT, OEx and KD mice *A–C*, representative RR‐interval plots of WT (*A*), OEx (*B*) and KD mice (*C*) during the low activity period. *D–F*, dot plot of the HR (beats min^−1^) of WT (*D*), OEx (*E*) and KD mice (*F*). The dotted arrows indicate the injection of atropine and propranolol. *G–I*, poincaré plots of the RR intervals. WT (*G*), OEx (*H*) and KD mice (*I*) during low activity periods. *J–L*, representative power spectral density plots. WT (*J*), OEx (*K*) and KD mice (*L*). Note that range of the ordinate axis of *L* is enlarged. The spectrum was divided into 3 frequency ranges. HF, high frequency band (1.5–4 Hz); LF, low frequency band (0.5–1.5 Hz); VLF, very low frequency band (0–0.5 Hz).

We plotted RR intervals for 1 min at the most frequently observed values of the HR during the low activity period: WT, ∼440–460 beats min^−1^; OEx, ∼460–480 beats min^−1^; KD, ∼400–420 beats min^−1^, as indicated by the peak of the HR histograms (see Fig. 4 *C* and *D*). As is evident from Fig. 5 *A*–*C*, the baseline fluctuation of the RR interval was clearly smaller in OEx mice and larger in KD mice compared with the WTs. In all strains of mice, HRV was completely suppressed by an injection of atropine (0.5 mg kg^−1^, i.p.) and propranolol (5 mg kg^−1^, i.p.), indicating that the fluctuation was due to autonomic regulation of the SAN (Fig. 5 *D*–*F*).

**Figure 6:**
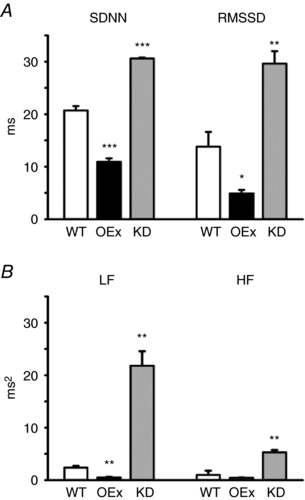
Summary of time domain analysis and frequency domain analysis *A*, time domain RR interval variability parameters of WT, OEx and KD mice. The standard deviation of all normal RR intervals in the sinus rhythm (SDNN) and the square root of the mean of the squared differences between successive normal RR intervals (RMSSD) were calculated from a 2 h recording during the low activity period. *B*, frequency domain analysis of the heart rate variability in WT, OEx and KD mice. HRV was calculated during the low activity period. HF, high frequency band (1.5–4 Hz); LF, low frequency band (0.5–1.5 Hz); VLF, very low frequency band (0–0.5 Hz). ^*^ *P *< 0.05, ^**^ *P *< 0.01, ^***^ *P *< 0.001, *n* = 3 in each group.

The difference in the HRV was more clearly demonstrated in a Poincaré plot of successive RR intervals. Fig. 5 *G* shows an oval distribution pattern recorded in WTs as the control. As shown in Fig. 5 *H*, the plot of OEx mice also demonstrated an oval pattern, although the area of dispersion was smaller than the control plot, indicating that the beat‐to‐beat fluctuation was reduced in OEx mice. In accordance with this, the standard deviation of all RR intervals (SDNN) and the root mean square of the difference of successive RR intervals (RMSSD) were significantly decreased in OEx mice (Fig. 6 *A*). In contrast, the Poincaré plot of KD mice shown in Fig. 5 *I* demonstrated a widely spread comet pattern. Similar findings were also reported in patients carrying HCN4 mutations (Schweizer *et al*. 2010, 2014).

We next carried out frequency domain analysis of the HRV. As shown in Fig. 5 *J*–*L*, we divided the HRV spectra into three frequency ranges: high frequency (HF; 1.5–4 Hz), low frequency (LF; 0.4–1.5 Hz) and very low frequency (VLF; 0–0.4 Hz). In all frequencies, the power was drastically decreased in OEx mice (Fig. 5 *K*), whereas it was increased in KD mice (Fig. 5 *L*). These results are summarized in Fig. 6 *B*. Taken together, our findings strongly suggest that the response of the SAN to parasympathetic nerve input is modified in both OEx and KD mice.

### Genetic manipulation of HCN4 expression modified bradycardia induced by vagus nerve stimulation

In heart‐specific HCN4‐knockout mice, the bradycardia induced by carbachol injection was reportedly enhanced (Herrmann *et al*. 2007; Hoesl *et al*. 2008). However, it was reported that pharmacological effects on cardiac electrical activity did not fully mimic the effects of the direct autonomic nerve stimulations (Mantravadi *et al*. 2007). Therefore, we next stimulated the right cervical vagus nerve (CVN) with bipolar electrodes, and evaluated the contribution of HCN4 in the parasympathetic response.

**Figure 7:**
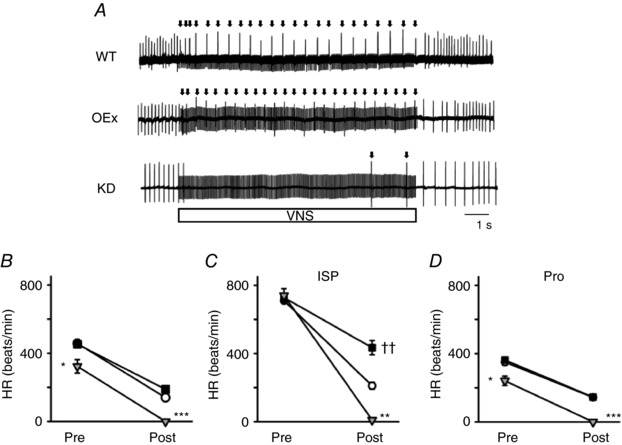
Reduction of the HR induced by cervical vagus nerve stimulation (CVNS) *A*, representative ECG traces of WT, OEx and KD mice pre/post CVNS. The box indicates the duration of CVNS. Note that electrical artefacts were recorded in ECGs during this period. The arrows indicate R waves overlapping the artefacts. *B*, summary of the HR reduction induced by CVNS. ○, WT (*n* = 4); ■, OEx (*n* = 4); ▽, KD (*n* = 4). *C*, HR reduction by CVNS in the presence of ISP. Symbols are the same as in *B*. *D*, HR reduction by CVNS in the presence of propranolol (5 mg kg^−1^). ^*^ *P* < 0.05, ^**, ††^ *P* < 0.01, ^***^ *P *< 0.001.

In WTs, we stimulated CVN (10 V at 20 Hz), so that an ∼50% reduction of HR was induced (top trace, Fig. 7 *A*). We then stimulated the CVN of OEx and KD mice using the same conditions. The magnitude of bradycardia was smaller in the OEx mice but was not significantly different from the WTs. In KD mice, however, CVN stimulation induced complete sinus pause, and the recovery from sinus pause was delayed after the termination of electrical stimulation (bottom trace, Fig. 7 *A*). In addition, the bradycardia response was completely inhibited by a pre‐injection of atropine (i.p. 0.5 mg kg^−1^) in all strains of mice. The basal HR of KD mice were significantly lower than those of WT and OEx mice under general anaesthesia (Fig. 7 *B*; 323.1 ± 39.3 *vs*. 457.2 ± 25.9 beats min^−1^).

However, β‐adrenergic acceleration of HR was maintained in KD mice; after injection of isoproterenol (ISP; i.p. 0.2 mg kg^−1^), the HR of WT, KD and OEx mice was not significantly different, as shown in Fig. 6 *C*. Under this condition, CVN stimulation revealed markedly different responses in the three strains of mice: WT showed a 70.3 ± 2.9% decrease in HR (710.0 ± 11.3 to 210.8 ± 20.3 beats min^−1^), whereas in OEx mice, the magnitude of bradycardia was significantly smaller (40.7 ± 4.5%; 728.0 ± 20 to 434.0 ± 41.7 beats min^−1^). Notably, CVN stimulation induced complete sinus pause in KD mice, even in this condition.

In addition to the direct effect on SAN function via the M2 muscarinic receptor, the parasympathetic nerve possesses the accentuated antagonism on the β‐adrenergic effect. Because the sympathetic nerve possesses background activity, we applied propranolol (5 mg kg^−1^), and performed CVN stimulation to examine the direct effect of the parasympathetic stimulation. As shown in Fig. 7 *D*, propranolol slightly reduced control HRs in all strains. CVN stimulation induced complete sinus pause in KD mice, whereas WT and OEx mice showed similar magnitudes of bradycardia.

These results suggested that an appropriate level of HCN4 expression is mandatory to maintain normal parasympathetic response of SAN, acting as a ‘limiter’ for bradycardia induced by potent parasympathetic nerve activity.

### Effect of acetylcholine on the spontaneous action potentials in PMCs isolated from WT, OEx and KD mice

**Figure 8:**
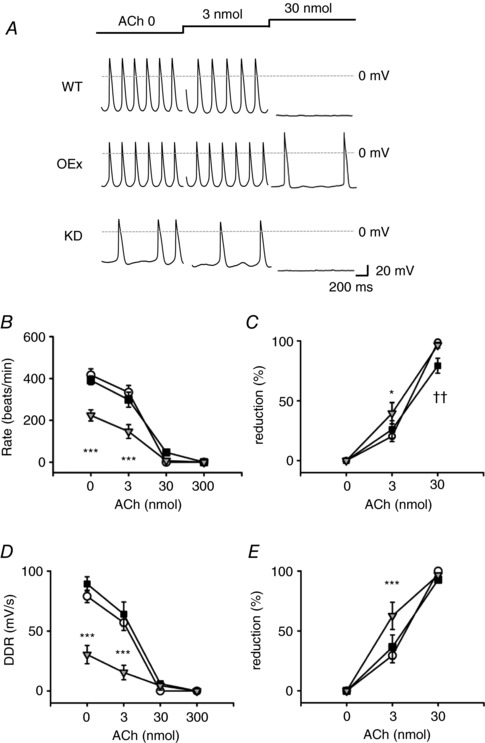
Effects of ACh on the SAPs of single PMCs isolated from WT, OEx and KD mice *A*, the effects of the cumulative application of ACh on SAPs of PMCs isolated from WT (top), OEx (middle) and KD (bottom) mice. *B–E*, the relationship between ACh concentration and FR (beats min^−1^) (*B*), percentage reduction of FR (*C*), DDR (mV s^−1^) (*D*) and percentage reduction of DDR (*E*). ○, WT (*n* = 11); ■, OEx (*n* = 11); ▽, KD (*n* = 11). Percentage reduction was calculated by normalizing each value by that in 0 nmol ACh. ^*^ *P *< 0.05, ††*P *< 0.01, ^***^ *P *< 0.001.

The effects of accentuated antagonism of acetylcholine (ACh) were reportedly complex even in an isolated heart preparation, because it contains sympathetic nerve endings and the parasympathetic ganglions (Cuevas *et al*. 1997; Brack *et al*. 2004; Merriam *et al*. 2004). In order to exclude the neural factors, we isolated single PMCs from the SAN, and recorded spontaneous action potentials (SAPs) of PMCs. Under this condition, we compared the negative chronotropic effect of ACh in the three strains of mutant mice (Fig. 8 *A*). In this experiment, the majority of PMCs isolated from KD mice showed irregular beating. We also selected PMCs based on their morphology and motility, excluding quiescent PMCs.

As summarized in Fig. 8 *B*, the firing rates (FR) were significantly slower in KD mice compared with the WTs in the control condition. The FR was not significantly different between OEx and WT mice, consistent with HR measurements *in vivo* (Fig. 7 *B*). ACh slowed FR in a dose‐dependent manner (Fig. 8 *C*). At 3 nmol, PMCs of KD mice showed a significantly larger reduction in FR (KD *vs*. WT; 39.6 ± 8.8% *vs*. 20.4 ± 4.4%). At 30 nmol, SAP was completely suppressed in the PMCs of WT and KD mice, whereas the PMCs of OEx mice still showed low frequency firing (OEx, 80.4 ± 22.2 beats min^−1^ *vs*. WT, 4.5 ± 4.5 beats min^−1^; KD, 0 beats min^−1^).

The effects of ACh on diastolic depolarization rates (DDR) of SAPs are summarized in Fig. 8 *D* and *E*. In the absence of ACh, the DDR was slightly higher in OEx than in WT mice, although this difference was not statistically significant. Consistent with the firing rate, the DDR of KD myocytes was significantly lower than those of the WTs at 0, 3 and 30 nmol (Fig. 8 *E* and *F*). These results indicated that the ACh response of single PMCs was enhanced in the KD mice but attenuated in the OEx mice.

**Figure 9:**
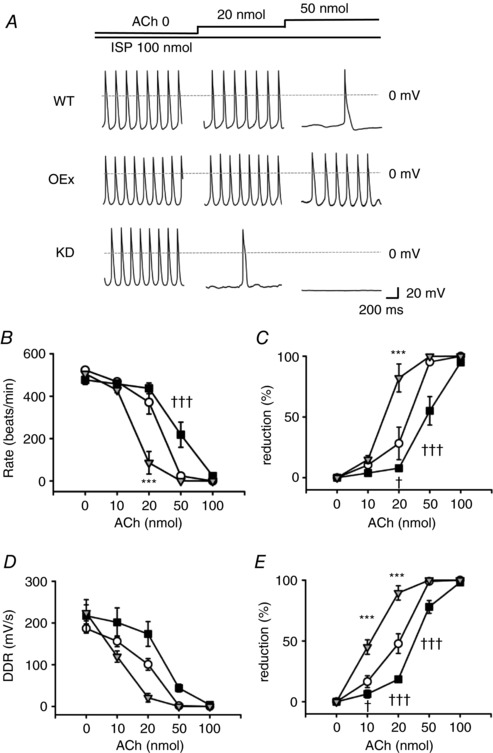
Effects of ACh with background ISP stimulation on the SAPs of single PMCs isolated from WT, OEx and KD mice *A*, the effects of the cumulative application of ACh in the presence of 100 nmol ISP. *B*–*E*, the relationship between ACh concentration and FR (beats min^−1^) (*B*), percentage reduction of FR (*C*), DDR (mV s^−1^) (*D*) and percentage reduction of DDR (*E*), in the presence of ISP. Percentage reduction was calculated by normalizing each value by that in 0 nmol ACh. ○, WT (*n* = 7); ■, OEx (*n* = 8); ▽, KD (*n* = 8), ^*, †^ *P *< 0.05, ^***, †††^ *P *< 0.001.

We next examined the accentuated antagonism of ACh in single PMCs. As shown in Fig. 9 *A*, we first applied 100 nmol ISP to the PMCs isolated from WT, OEx and KD mice. Notably, PMCs of KD mice showed stable SAPs in the presence of β‐adrenergic stimulation, which well agreed with the previous reports (Herrmann *et al*. 2007). The control FR was not significantly different between WT, OEx and KD mice. In contrast, the magnitudes of ACh effects were profoundly affected by the difference of expression levels of HCN4 (Fig. 9 *B* and *C*). At 20 nmol ACh, the PMCs of KD mice showed a significantly larger reduction in FR (KD *vs*. WT; 82.1 ± 7.1% *vs*. 28.2 ± 7.1%), whereas in the PMCs of OEx, the inhibition was significantly smaller (OEx, 7.8 ± 4.2%). At 50 nmol, the SAP was almost completely suppressed in the PMCs of WT and KD mice (WT, 95.2 ± 5.1%; KD, 99.9 ± 4.4%), whereas the magnitude of the inhibition was 55.1 ± 5.1% in the PMCs of OEx mice. Similar tendencies were found in the effects of ACh on DDR (Fig. 9 *D* and *E*).

### The effects of ACh on *I*_f_ current

**Figure 10:**
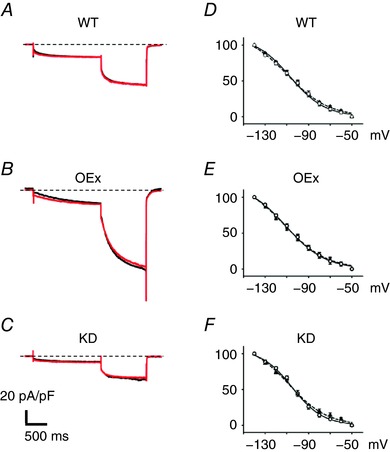
The effects of ACh on *I*_f_ currents of WT, OEx and KD PMCs *A–C*, *I*_f_ was activated by 2‐step voltage pulses to −100 mV and −150 mV. The holding potential was −50 mV. *A*, WT; *B*, OEx; *C*, KD. The black traces indicate *I*_f_ in the control condition, and the red traces, in the presence of 300 nmol ACh. *D–F*, the voltage‐dependent activation curves of *I*_f_ in WT (*D*), OEx (*E*) and KD mice (*F*). ○, control; ▴, in the presence of 300 nmol ACh (*n* = 6 in each experiment). The values of *V*_1/2_ (control *vs*. ACh) were: WT, −95.2 ± 3.2 *vs*. −98.7 ± 2.9 mV; OEx, −97.5 ± 1.9 *vs*. −101.5 ± 1.8 mV; KD, −93.3 ± 1.6 *vs*. −99.1 ± 1.6 mV.

In rabbit PMCs, ACh reportedly inhibited *I*_f_ in the basal condition, and thereby slowed the SAP (DiFrancesco *et al*. 1989). We therefore examined the effects of 300 nmol ACh, which completely inhibit SAPs, on *I*_f_ currents in our system. As evident from Fig. 10 *A*–*C*, traces of *I*_f_ in the presence (red line) and absence (black line) of ACh were almost superimposable. As shown in Fig. 10 *D*–*F*, the voltage‐dependent activation curves were not significantly different in the presence (filled triangles) and absence (open circles) of ACh, in all strains of transgenic mice. A similar finding was also reported in pregnant mice (El Khoury *et al*. 2013). Unlike in rabbit SAN, these results may also support the notion that *I*_f_ current did not expedite, but attenuated the parasympathetic response of murine SAN.

## Discussion

The present study demonstrated for the first time, that phenotypic changes in SAN function induced by conditional overexpression of HCN4 channels; genetic overexpression of HCN4 attenuated the parasympathetic response of the SAN and reduced the HRV. Furthermore, we have provided direct evidence that HCN4 channels prevent excessive bradycardia induced by potent parasympathetic nerve activity. We concluded that HCN4 channels played a protective role in parasympathetic regulation. The cAMP‐dependent activation of HCN4 channels appeared to enhance this protective effect, rather than expediting sympathetic response of the SAN.

In this study, HCN4 channels were globally overexpressed or knocked‐down, including in the central nervous system (CNS). At present, the physiological functions of HCN4 in the CNS remain unclear (Biel *et al*. 2009). As shown in the present study, the modifications of parasympathetic response observed in single PMCs were consistent with the phenotypic changes *in vivo*. Therefore, it seems safe to conclude that alterations in HR regulation in OEx and KD mice are attributable to the SAN, rather than the CNS.

All members of the HCN channel family possess a cyclic nucleotide‐binding motif at their C‐terminus. During β‐adrenergic stimulation, cAMP binds to this motif and increases the amplitude of the *I*_f_ by shifting the voltage‐dependent activation curves of HCN2 and HCN4 in a positive direction (Biel *et al*. 2009; Liao *et al*. 2010; El Khoury *et al*. 2013). Due to this property, HCN channels have been thought to play a critical role in the positive chronotropic effect of β‐adrenergic stimulation (DiFrancesco, 2010) However, genetic ablation of cAMP sensitivity of HCN4 as well as transgenic overexpression of mutant HCN4 (HCN4‐573X‐Tg) that produces dominant negative suppression of cAMP sensitivity in heteromeric HCN channels did not inhibit β‐adrenergic acceleration of the HR (Harzheim *et al*. 2008; Alig *et al*. 2009). A similar phenotype was also reported in a patient carrying an HCN4 mutation that lacked cAMP‐sensitivity (Schweizer *et al*. 2010). A β‐adrenergic chronotropic effect may be mainly attributable to a Ca^2+^ clock mechanism and the up‐regulation of D‐ and L‐type Ca^2+^ channels (Lakatta *et al*. 2010; Mesirca *et al*. 2015).

In the SAN, the basal cAMP level may be high due to the ligand‐free constitutive activity of the β2‐adrenergic receptors that co‐localize with HCN4 channels (Zhou *et al*. 2000; Barbuti *et al*. 2007; Greene *et al*. 2012). Basal cAMP may be required for maintaining the basal function of HCN channels because sinus arrhythmia was observed in transgenic mice in which the *I*_f_ lacked cAMP‐sensitivity (Harzheim *et al*. 2008; Alig *et al*. 2009). Therefore, similar to ‘accentuated antagonism’, stimulation of muscarinic M2 receptors may regulate the *I*_f_ by inhibiting basal cAMP production via the β‐subunit of G_i_ proteins. In rabbit PMCs, low dose ACh reportedly decreased the amplitude of the *I*_f_ by shifting the activation curve in a negative direction in the basal condition (DiFrancesco *et al*. 1989). ACh also decreased the firing rate of SANs at a similar concentration. Therefore, inhibition of the *I*_f_, rather than activation of muscarinic K^+^ channels (I_KACh_), was suggested to play a pivotal role in bradycardia induced by M2 muscarinic receptors (DiFrancesco *et al*. 1989; DiFrancesco, 2010). However, in mouse PMCs, ACh reportedly did not inhibit the basal *I*_f_ (El Khoury *et al*. 2013). Likewise, we could not observe ACh‐induced inhibition of the *I*_f_ in the basal condition (Fig. 6). At present, we do not have a ready explanation for this difference; it might be due to species differences. In human, however, *I*_f_ seems to play similar a antagonistic role against the parasympathetic response: in the patients carrying a loss‐of‐function mutation of HCN4, it was reported that the RR‐interval was profoundly prolonged at night during which the parasympathetic nerve activity was higher (Schweizer *et al*. 2014). This finding agreed well with the HR histogram of HCN4‐KD mice obtained in the low activity period.

In a ligand‐free condition, spontaneous opening of I_KACh_ reportedly contributed to the background outward conductance of rabbit PMCs (Ito *et al*. 1994). Genetic ablation of GIRK4 successfully rescued SAN dysfunction in transgenic mice which express dominant negative non‐conductive HCN4 subunits in their hearts (HCN4‐AYA‐Tg) and in knockout mice lacking Cav1.3 (Mesirca *et al*. 2014, 2016). This finding was most likely due to correcting an imbalance between the outward and the inward currents underlying the pacemaker potential. The autonomic regulation of the HR was abnormal in GIRK4 knockout mice (GIRK4^−/−^) that lacked I_KACh_; HR variability in the HF and LF ranges was reduced in GIRK4^−/−^ mice (Wickman *et al*. 1998). As shown in the present study, we found that overexpression of HCN4 also decreased the HRV in all frequency ranges. Taken together, the activation of the *I*_f_ in the SAN appears to counteract *I*_KACh_, and therefore decreases the HRV by buffering the parasympathetic response.

In cardiac hypertrophy, expression of fetal type ion channels including HCN4 was reportedly reactivated in the ventricle (Kuwahara & Nakao, 2011). In the SAN, however, pathological remodelling of ion channels remains poorly understood. A recent study has reported that physical training down‐regulated HCN4 expression in the SAN and thereby induced bradycardia (D'Souza *et al*. 2014). Expression of HCN4 may be down‐regulated with age (Huang *et al*. 2016). The expression of ion channels in the SAN may also be regulated by circadian rhythms (Yaniv & Lakatta, 2015). In the present study, we could successfully visualize HCN4‐expressing cells with the luciferin–luciferase reaction, which is widely used for promoter assays. Therefore, HCN4^+/luc^ mice would be useful for quantitative evaluation of the remodelling of HCN4 expression in future studies.

In the cardiac conduction system (CCS), the phenotypic effects of genetic silencing of HCN4 appear controversial; atrio‐ventricular (AV) block was observed in heart‐specific ic‐HCN4‐KO mice and in HCN4‐AYA‐Tg mice (Baruscotti *et al*. 2011; Mesirca *et al*. 2014). The QRS complex was also prolonged in HCN4‐AYA‐Tg mice (Mesirca *et al*. 2014), although in other ic‐HCN4‐KO mice and HCN4‐KD mice, these parameters were not changed (Herrmann *et al*. 2007; Hoesl *et al*. 2008). Conversely, PR and QRS durations were not shortened in HCN4‐OEx mice (Fig. 2). It has recently been reported that tachycardia in pregnant mice was due to the increased expression of HCN2 in the SAN. The PR interval was also shorter in pregnant mice, although ion channel remodelling of the AV node has not yet been explored (El Khoury *et al*. 2013). The transgenic mouse overexpressing HCN2 specifically in the heart reportedly showed tachycardia, but PR and QRS durations were not shortened (Oshita *et al*. 2015). At present, the reasons for these discrepancies remain unclear; electrophysiological mechanisms regulating conduction velocity in the CCS should be explored in future research.

## Additional information

### Competing interests

All authors have no conflict of interests.

### Author contributions

M.T. conceived the study. Y.K., N.N. and M.T. designed the work. Y.K., M.I., T.I., H.K., N.M. and K.U. collected the data. Y.K. analysed the data. Y.K., N.N. and M.T. interpreted the data. Y.K. and M.T. drafted the article, and all the authors revised it critically. All authors have approved the final version of the manuscript and agree to be accountable for all aspects of the work. All persons designated as authors qualify for authorship, and all those who qualify for authorship are listed.

### Funding

This work was supported by a Grant‐in‐Aid for Scientific Research from the Japan Society for the Promotion of Science (JSPS KAKENHI Grant Numbers 16H05124 and 24300145).
